# Coexistent Eosinophilic Esophagitis and Dysplastic Barrett's Esophagus With Rapid Eosinophilic Infiltration of Neosquamous Mucosa After Radiofrequency Ablation

**DOI:** 10.14309/crj.0000000000001488

**Published:** 2024-08-29

**Authors:** Prince A. Ameyaw, David Parsons, Ali Mahmoud, Robert Marie, Anil Nagar, Harry R. Aslanian

**Affiliations:** 1 Department of Medicine, Bridgeport Hospital/Yale New Haven Health, Bridgeport, CT; 2 Section of Digestive Diseases, Yale School of Medicine, New Haven, CT; 3 Department of Pathology, Yale School of Medicine, New Haven, CT

**Keywords:** radiofrequency ablation, eosinophilic esophagitis, Barett's esophagus, dysplasia

## Abstract

The coexistence of eosinophilic esophagitis (EoE) and Barrett's esophagus (BE) is rare despite the known association of gastroesophageal reflux disease with both conditions. Radiofrequency ablation is an effective endoscopic eradication therapy in patients with dysplastic BE. However, the efficacy and outcomes of radiofrequency ablation in patients with concomitant EoE and BE are not well known. We report a case of rapid eosinophilic infiltration of the neosquamous mucosa after the complete eradication of long-segment dysplastic BE in a patient with coexisting BE and EoE.

## INTRODUCTION

Eosinophilic esophagitis (EoE) is defined by esophageal dysfunction with symptoms of dysphagia and heartburn, endoscopic findings of esophageal rings, strictures, furrows, and at least 15 eosinophils per high-power field on histology.^1^ Gastroesophageal reflux disease (GERD) may be associated with esophageal eosinophilia, particularly in the distal esophagus, and should be excluded.^2^ Although GERD may predispose to esophageal eosinophilia and the development of Barrett's esophagus (BE), associations between EoE and BE are uncommon.^2,3^ Treatment of dysplastic BE with radiofrequency ablation (RFA) has been shown to reduce progression to neoplasia.^4^ We present a rare case of rapid development of eosinophilic infiltration of the neosquamous epithelium after RFA in a patient with coexisting EoE and long-segment BE with dysplasia.

## CASE REPORT

A 44-year-old man initially presented with dysphagia to meat and heartburn. He reported no tobacco use or pharmacologic or environmental allergies. His physical examination was normal. Esophageal rings and mucosal furrows were found in the proximal and midesophagus on initial endoscopy, as well as a 9-mm midesophageal ringed stenosis dilated by scope passage. Biopsies revealed distal long-segment Barrett's with low-grade and focal high-grade dysplasia and increased intraepithelial eosinophils of 25–30/hpf in the proximal esophagus. He was maintained on a daily proton pump inhibitor (PPI) after a course of budesonide with symptomatic response.

**Figure 1.**
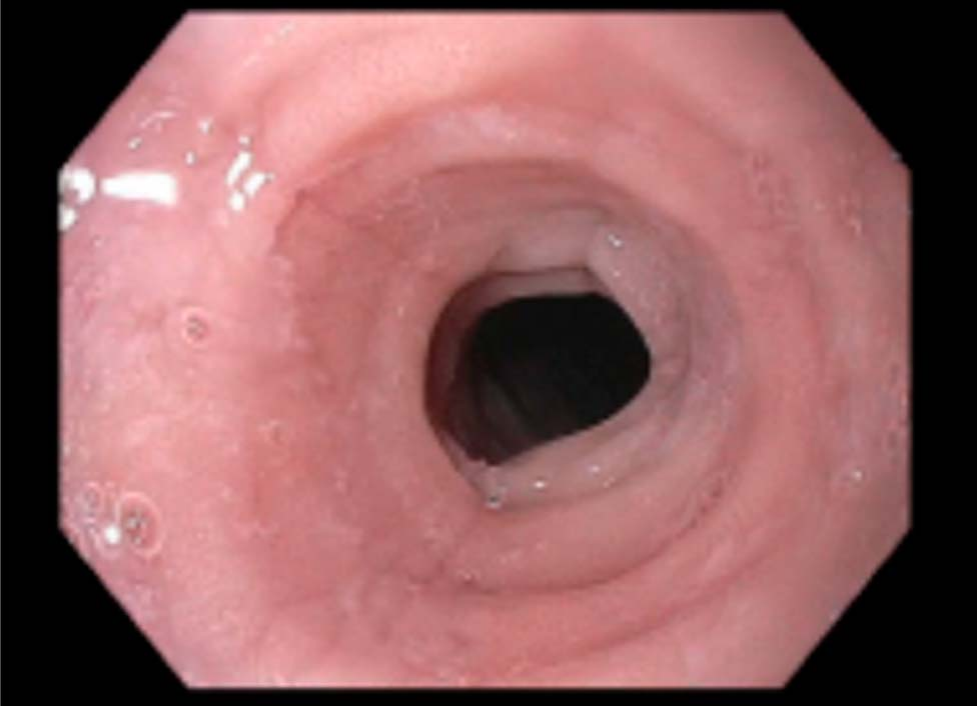
Endoscopic image showing circumferential mucosal rings and stenosis in proximal and midesophagus.

**Figure 2.**
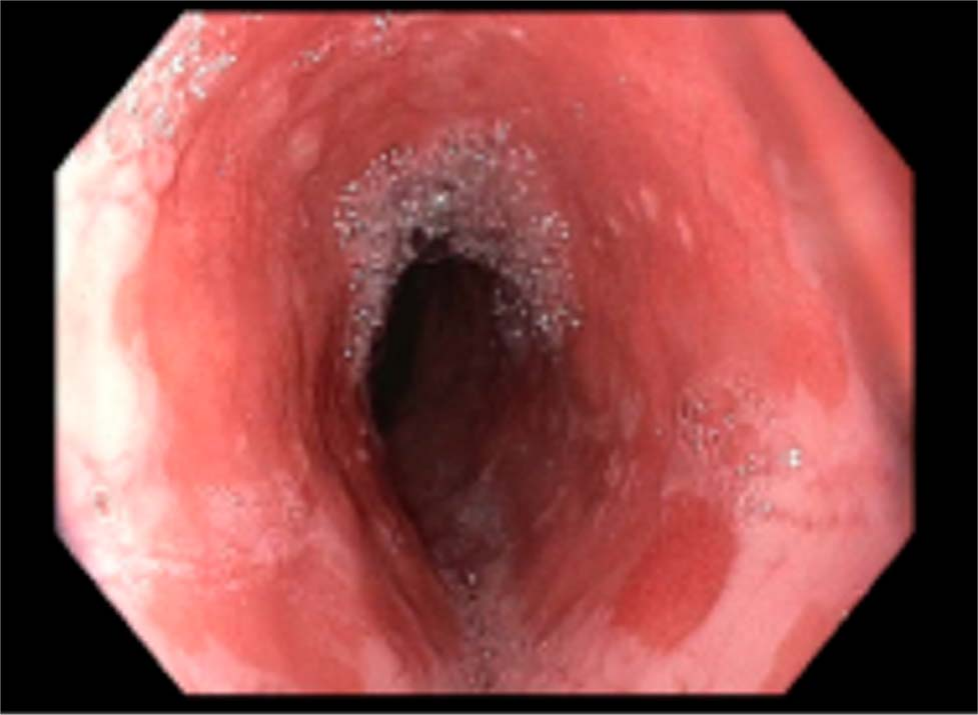
Endoscopic image showing salmon-colored mucosa of the distal esophagus consistent with Barrett's esophagus.

**Figure 3.**
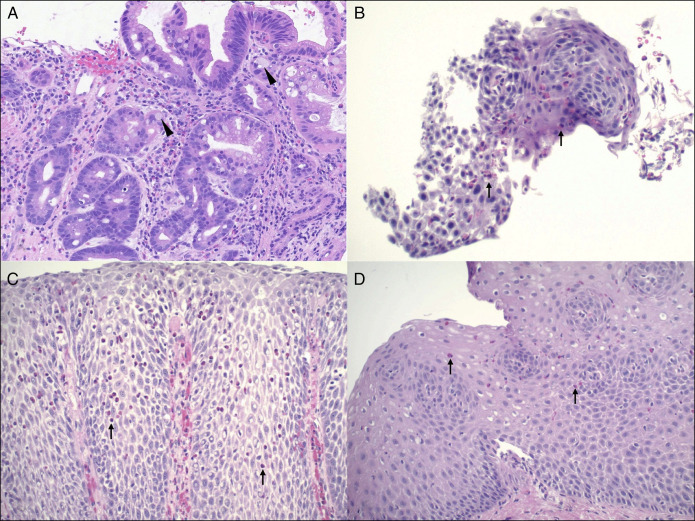
(A) Initial biopsies from the distal esophagus revealed intestinal metaplasia with numerous goblet cells (arrowheads), in addition the columnar cells show enlarged, mildly atypical nuclei both in the gland base and extending to the surface, characteristic of low-grade dysplasia. Magnification: 200×. (B) The proximal esophageal mucosa at 24 cm revealed eosinophil-rich esophagitis with eosinophils numbering >25/high-power field (arrows) and hyperplasia of the basal zone. No lamina propria was present in the biopsies to evaluate subepithelial fibrosis. (C) After radiofrequency ablation, the gastroesophageal junction revealed re-epithelialization by squamous epithelium with eosinophil-rich esophagitis. (D) Concomitant proximal esophageal mucosa (which had not undergone ablation) also continued to show eosinophilic esophagitis. Basal zone hyperplasia, subepithelial fibrosis, and focal eosinophilic microabscesses were noted in several biopsies.

Endoscopy 3 months later revealed circumferential mucosal rings with linear furrows and benign-appearing stenoses in the upper and middle third of the esophagus (Figure 1). Salmon-colored mucosa was seen from 29 to 40 cm, consistent with long-segment circumferential BE (C10M11) (Figure 2). Histopathology revealed more than 50 eosinophils/hpf in the proximal squamous mucosa and BE with low-grade dysplasia bordering on focal high-grade dysplasia without increased eosinophils in the Barrett's segment (Figure 3). RFA of the long-segment Barrett's dysplasia was performed, and PPI therapy was increased to twice daily.

He had a good response to RFA with the eradication of 75% of the Barrett's segment after 3 RFA treatments with a focal RFA catheter (Halo 90 Barrx; Medtronic, Minneapolis, MN). Repeat biopsy 8 months after initial RFA, revealed a diffuse eosinophilic infiltration (peak count > 50/hpf) of the nondysplastic neosquamous mucosa within the previous dysplastic Barrett's segment and persistence of EoE (peak eosinophil count > 25/hpf) in the proximal esophagus. Despite these new changes of diffuse distal esophageal eosinophilia, he denied any symptoms of dysphagia, heartburn, or chest pain. Medical management was escalated with dupilumab 300 mg weekly because of the persistence of EoE on biopsy.

An additional RFA treatment achieved complete eradication of the long-segment Barrett's, with 4 treatments performed at average follow-up intervals of 4 months. Biopsy from the surveillance endoscopy 4 months after the last RFA revealed diffuse EoE involving the entire length of the neosquamous mucosa with a peak eosinophil count of >50/hpf, along with persistent eosinophilia of the EoE present in the upper esophagus and midesophagus. The patient did not develop any symptoms of dysphagia within 18 months of follow-up, despite the development of EoE in the neosquamous mucosa of the distal esophagus after ablation.

## DISCUSSION

BE, in addition to esophageal eosinophilia, is associated with GERD. The coexistence of EoE and dysplastic BE, however, remains rare, with only a few reported cases.^2,5,6^ We present a rare case of long-segment, dysplastic BE without eosinophilia, responsive to RFA, with the development of EoE within the neosquamous (post-treatment) mucosa within 4 months of RFA treatment.

Untreated Barrett's dysplasia is associated with a lifetime esophageal adenocarcinoma risk of up to 5%.^7^ RFA has emerged as the primary endoscopic eradication therapy for dysplastic BE with complete eradication rates of >90% in the AIM dysplasia trial and subsequent studies.^8–10^ RFA uses thermal energy derived from electrical current to induce superficial tissue necrosis, which allows for neosquamous epithelium regeneration and minimizes the risks of stricture formation.^11^

Rates of the development of eosinophilia after RFA in patients without EoE have been reported in 2 studies at 2.7% after 1 year in 140 patients and 9% (after RFA or cryotherapy) for dysplastic BE after 3 years with a positive linear correlation between Barrett's esophagus segment length and postablation eosinophilia.^12,13^ The median postablation eosinophil counts were 15 and 39/hpf (range 5–103).^12,13^ None of the patients from the above studies had a documented diagnosis of EoE before RFA. Halsey et al found a median eosinophil count of 1/hpf (range 0–14) in the Barrett's segment before ablation, although no pre-RFA esophageal eosinophilia was identified by Villa et al.^12,13^ In a retrospective study by Owens et al, patients who had undergone ablation (RFA or photodynamic therapy) for Barrett's dysplasia were followed with endoscopic biopsies of the neosquamous mucosa after ablation. 3.4% of the patients (13/385, all following photodynamic therapy) had increased eosinophils (≥30/hpf) in the neosquamous mucosa with onset between 83 and 692 days after ablation. There was no dysphagia attributed to eosinophilia or endoscopic signs of EoE. None of the patients had a diagnosis of EoE before ablation.^14^ This is the first reported case of a patient with known EoE and BE found to have EoE extending into the neosquamous esophagus after RFA and in which the timeframe of eosinophilic infiltration has been documented.

Chronic inflammation because of repetitive ablation-induced injury, increased sensitivity of the ablated epithelium to ingested antigens, and ongoing GERD have been proposed as possible etiologies of post-RFA esophageal eosinophilia.^13^ An increased incidence of proximal esophageal fibrostenotic disease was found on endoscopy in patients with coexistent EoE and BE (4.7% of 509 patients) along with a male preponderance, in data analyzed from the Swiss Eosinophilic Esophagitis Cohort Study.^15^ Consistent with the above study, our patient was male and had dysphagia because of esophageal stenoses in the proximal EoE segment, which responded well to dilation, PPI, and dupilumab. No eosinophilia was seen on biopsy of the Barrett's segment; however, eosinophilia within the post-RFA treatment neosquamous mucosa was seen to develop within 4 months after therapy. The development of eosinophilia in the distal esophagus was not associated with new-onset symptoms of esophageal dysfunction.

This case demonstrates a good response to RFA in a patient with concomitant EoE and long-segment dysplastic Barrett's and provides interesting insights into the pathophysiology of EoE because high levels of eosinophilia were seen to develop within the neosquamous mucosa within 4 months after RFA ablation, despite the use of high-dose PPI therapy and the absence of significant reflux esophagitis. Further data on the efficacy and outcomes of RFA in patients with coexistent EoE and dysplastic BE is required. In addition, further study of the clinical course and potential significance of post-RFA eosinophilia in patients with and without concomitant EoE may provide additional insights into the pathophysiology of EoE and aid in risk stratification and guide postablation endoscopic surveillance.

## DISCLOSURES

Author contributions: P.A. Ameyaw performed the initial chart and literature review to produce the draft manuscript. D. Parsons led concept designing and literature review. A. Mahmoud and R. Marie were responsible for preparing the esophageal microscopy slides and describing the legends. A. Nagar and H.R. Aslanian performed a revision of the final manuscript. H.R. Aslanian was the supervising clinician, participated in concept design, and produced the final manuscript. P.A. Ameyaw is the article guarantor.

Financial disclosures: H.R. Aslanian is a consultant, at Boston Scientific and Olympus.

Informed consent was obtained for this case report.
